# Reductive radical-polar crossover: traditional electrophiles in modern radical reactions

**DOI:** 10.1039/c9sc03359a

**Published:** 2019-08-16

**Authors:** Lena Pitzer, J. Luca Schwarz, Frank Glorius

**Affiliations:** a Organisch-Chemisches Institut , Westfälische Wilhelms-Universität Münster , Corrensstraße 40 , 48149 Münster , Germany . Email: glorius@uni-muenster.de

## Abstract

The concept of reductive radical-polar crossover (RRPCO) reactions has recently emerged as a valuable and powerful tool to overcome limitations of both radical and traditional polar chemistry. Especially in case of additions to carbonyl compounds, the synergy of radical and polar pathways is of great advantage since it enables the use of traditional carbonyl electrophiles in radical reactions. The most recent and synthetically important transformations following this line are summarised in the first part of this review. The second part deals with transformations, in which the concept of RRPCO promotes the usage of alkyl halides as electrophiles in radical reactions.

## Abstract 2

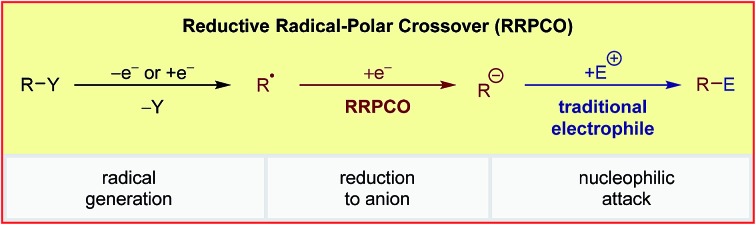
The concept of reductive radical-polar crossover (RRPCO) reactions has recently emerged as a valuable and powerful tool to overcome limitations of both radical and traditional polar chemistry.

## 1. Introduction

**Scheme 1:**
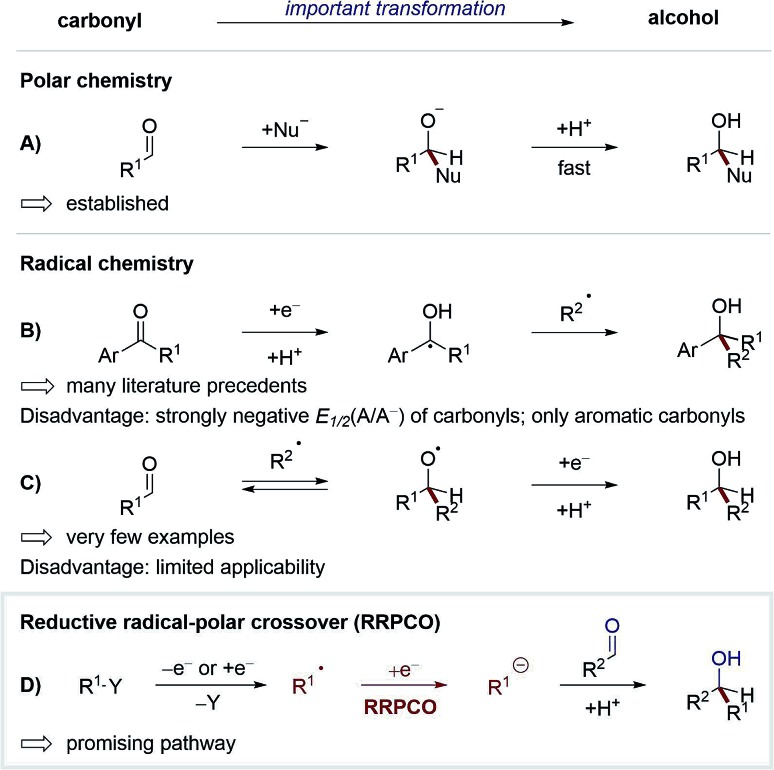
Strategies to convert carbonyls to alcohols.

One of the most studied and fundamental transformations of organic chemistry is the addition of nucleophiles to carbonyls for the synthesis of alcohols. This transformation can be performed in a multitude of ways with high levels of diastereo- and enantioselectivity. While these reactions have been extensively developed in polar chemistry, radical pathways for this transformation remain underexplored. The development of such radical reactions would enable a wider variety of nucleophiles to be used, and hence generate valuable new product motifs. Furthermore, in combination with visible-light-mediated photochemistry, radical chemistry could be used to promote elusive transformations with high levels of functional group tolerance.^1^ Until recently, only two strategies existed to convert carbonyls to alcohols *via* visible-light-mediated radical pathways (Scheme 1A–C). In the first strategy, carbonyls are reduced to form stabilised ketyl-radicals, which due to the persistent-radical-effect can be coupled selectively with transient radicals to yield alcohols as products.^2^ Whilst numerous methods have been developed according to this strategy,^3^ some disadvantages limit its generalisation. For example, carbonyl compounds typically exhibit a strongly negative redox potential (*E*_1/2_(acetophenone) = –2.11 V *vs.* SCE),^4^ thus their reduction requires the use of strongly reducing reagents/catalysts, which limits the functional group tolerance of these methods. Additionally, the scope of this strategy is limited to aromatic ketones or aldehydes, since selective radical-radical-cross-coupling can only be achieved if the lifetime of the ketyl radical is sufficiently long, which typically requires mesomeric stabilisation.

**Scheme 2:**
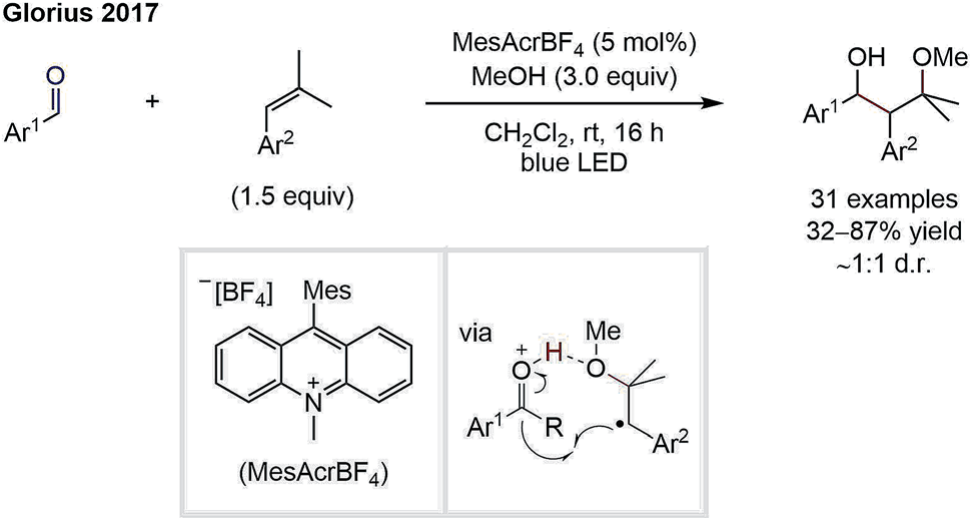
Approach to use carbonyls as intermolecular radical acceptors by Glorius.^6^

In the second strategy, carbonyls are used as intermolecular radical acceptors (Scheme 1C). Based on seminal reports from Clerici and Porta from the 1980's,^5^ this strategy was recently reintroduced by the Glorius group.^6^ The main challenge of this approach is the instability of the intermediary formed alkoxy radical, which normally decays directly *via* C–C-β-scission. Here, Glorius *et al.* showed that this obstacle could be overcome by adding alkyl radicals to protonated carbonyls to form alkoxy radical cations intermediates, which are less prone to C–C-β-scission and can be more easily reduced than alkoxy radicals (Scheme 2). It should be noted that diastereo- and enantioselective variants of this strategy remain elusive.

Based on the weaknesses of the aforementioned strategies, a new way to use carbonyls as acceptors in radical chemistry has recently attained broad interest. This concept combines radical and polar chemistry, a so-called reductive radical-polar crossover (RRPCO) (Scheme 1D). In this approach, a suitable alkyl radical is converted into a nucleophile either through reduction or capture by a transition metal complex. These nucleophilic species can then react with carbonyl compounds as traditional electrophiles. By employing this strategy the mildness and broad functional group tolerance of radical chemistry is combined with the better selectivity and control of polar chemistry. To date, only a few methods of RRPCO are known, of which selected examples are highlighted in this minireview. Aside from carbonyl electrophiles, nucleophilic substitutions with alkyl halide or tosylate electrophiles have also been described and will be discussed in the second part of the minireview.

## 2. Reductive radical-polar crossover using carbonyls as electrophiles

### 2.1 Visible-light photoredox mediated transformations

**Scheme 3:**
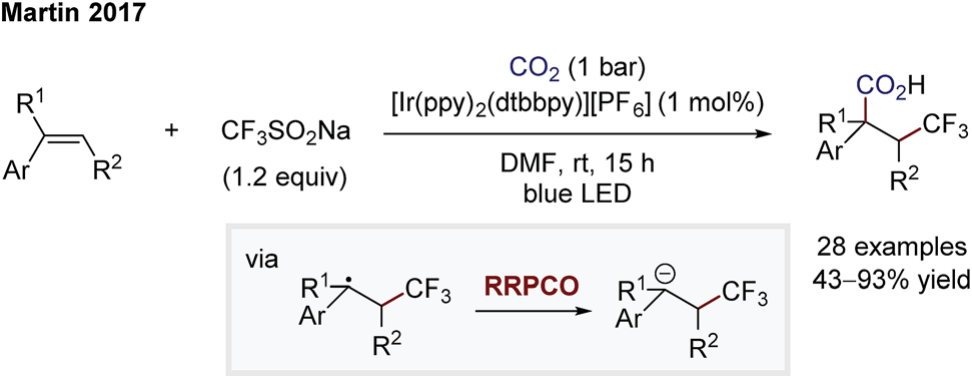
Photoredox-mediated dicarbofunctionalization of styrenes by Martin.^8^

Whilst the groups of Ryu and Sonoda were the first to describe the RRPCO concept in the 1990's,^7^ this strategy has recently received renewed interest through its combination with visible-light photoredox catalysis. This is owing to the unique reactivity of photoredox catalysts, which are able to facilitate the two electron transfer steps required – the first to generate a radical from a radical precursor, and the second, to reduce the generated radical to form a nucleophile (Scheme 1D). The first group to demonstrate the high synthetic potential of RRPCOs by means of visible-light photoredox catalysis was the Martin group in 2017 (Scheme 3).^8^ Here, an Ir-photocatalyst ([Ir(ppy)_2_(dtbbpy)][PF_6_]; ppy: 2-phenylpyridine, dtbbpy: 4,4′-di-*tert*-butyl-2,2′-bipyridine) was used to generate trifluoromethyl radicals, which were added to substituted styrenes to form benzyl radicals. These stabilised benzyl radical intermediates were then reduced by the photocatalyst to form carbanions, which were trapped with carbon dioxide to deliver the carboxylic acid products. Evidence for the presence of an anionic species in this reaction was obtained by trapping with deuterium oxide instead of carbon dioxide. Furthermore, thermodynamic considerations of the two electron transfer steps by redox potential comparison also supported this mechanistic proposal. This challenging dicarbofunctionalization reaction highlighted the ability of this powerful synergy between radical and polar chemistry to generate valuable building blocks from simple reagents.

**Scheme 4:**
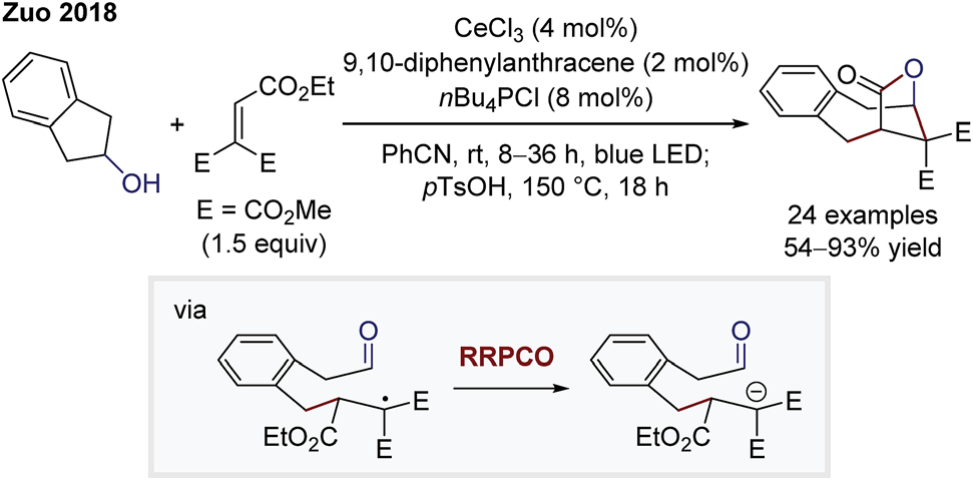
Cerium- and photoredox-mediated bridged lactonisation by Zuo.^9^

In 2018, the group of Zhiwei Zuo reported on a dual cerium- and photoredox-catalysed bridged lactone formation starting from cycloalkanols and Michael acceptors (Scheme 4).^9^ At first, the alkoxy radical is generated through a cerium(iv)-mediated ligand-to-metal charge transfer. Owing to the instability of such radicals, a C–C-β-scission generates a more stabilised alkyl radical. At this point, the photocatalyst-induced RRPCO takes place, which is followed by a nucleophilic addition of the generated anion to the pendant aldehyde moiety. Lactone formation is caused after ester hydrolysis, delivering the bridged product motifs. Once again, the interplay of radical and polar pathways results in the formation of complex products from simple starting materials in a single step.

**Scheme 5:**
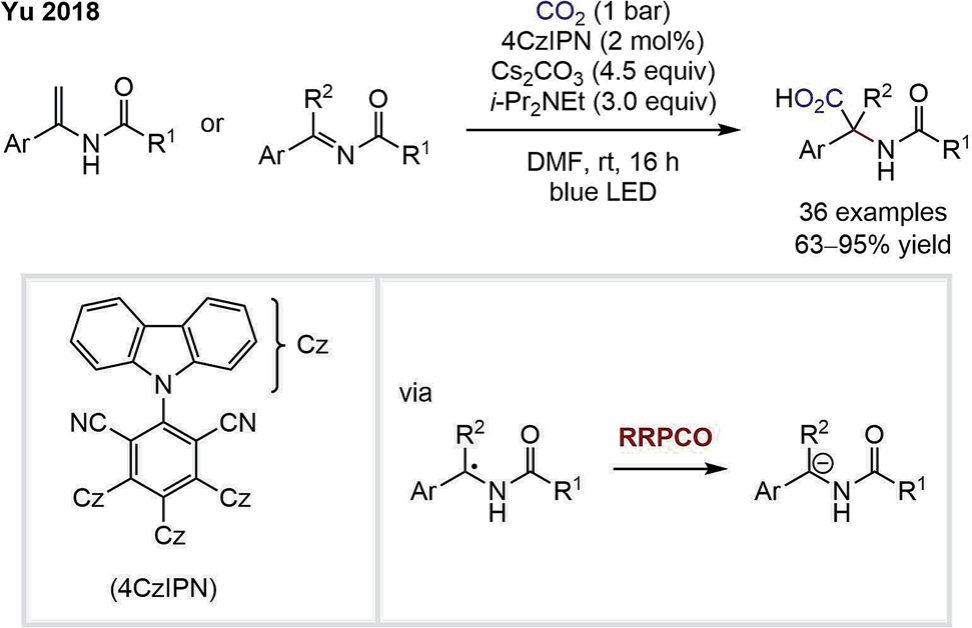
α-Carboxylation of enamindes and imines by Yu.^10^

In 2018, the group of Da-Gang Yu reported a highly chemo- and regioselective visible-light-mediated carboxylation of enamides and imines for the synthesis of α,α-disubstituted α-amino acids (Scheme 5).^10^ An α-amino anion is formed *via* two consecutive reductions of an imine intermediate by the excited state photocatalyst. This anion then adds to carbon dioxide to form the α-amino acid product. The existence of a carbanion intermediate was suggested by deuteration experiments and trapping with other electrophiles, such as carbon disulfide or isothiocyanates. The high regioselectivity for the α-carboxylation in this transformation is remarkable and is complementary to classical carboxylations of enamides that typically proceed in the β-position.

**Scheme 6:**
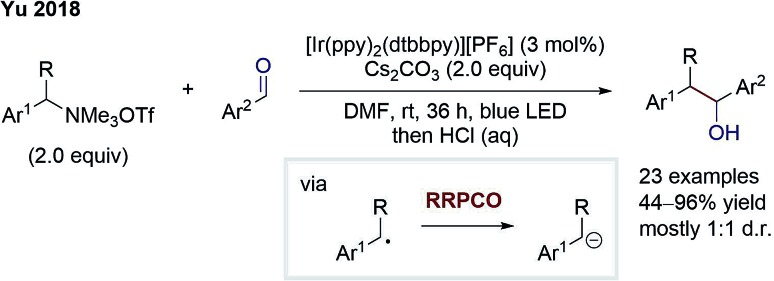
Cross-electrophile coupling by Yu.^11^

Later in 2018, the group of Yu also disclosed an elegant external-reductant-free cross-electrophile coupling between tetraalkylammonium salts and carbonyls (Scheme 6).^11^ Mechanistically, the reaction proceeds as follows: a benzylic radical is generated from the ammonium salt by an excited Ir-photocatalyst-mediated reductive deamination. Next, this benzylic radical is reduced by a second excited state Ir-species. Subsequently, the formed benzyl anion adds to the carbonyl compound – mostly benzaldehydes – to give the desired alcohol product. Further, Yu and co-workers could also switch to carbon dioxide as the electrophile to generate carboxylic acids as products. The benzylic anion intermediate was suggested by deuteration experiments. Almost complete deuterium incorporation was observed when adding deuterium oxide to the reaction. In contrast to this, DMF-*d*_7_ did not cause any deuteration, contradicting a putative hydrogen atom transfer (HAT) step. In conclusion, this cross-electrophile coupling underlines the high potential of RRPCO, overcoming traditional philicity limitations.

**Scheme 7:**
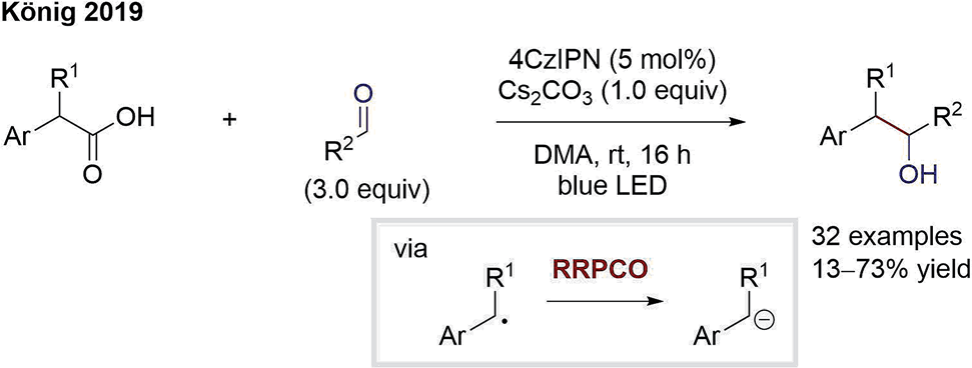
Benzylation of aliphatic aldehydes by König.^12^

The group of König reported on a similar transformation (Scheme 7).^12^ Here, radical formation proceeds *via* 4CzIPN (2,4,5,6-tetrakis(carbazol-9-yl)-1,3-dicyanobenzene)-mediated oxidation of benzyl carboxylates followed by rapid decarboxylation. The stabilised benzylic radical is converted to an anion through electron transfer from the reduced photocatalyst. After the RRPCO, nucleophilic addition to aliphatic aldehydes and ketones takes place, generating alcohols as products. Again, deuterium incorporation by addition of deuterium oxide signifies the intermediary formation of anions. The successful addition to aliphatic carbonyl compounds instead of aromatic ones reveals the improvement gained through RRPCO in comparison to radical chemistry alone, where to date only aromatic carbonyls could be used as acceptors.

**Scheme 8:**
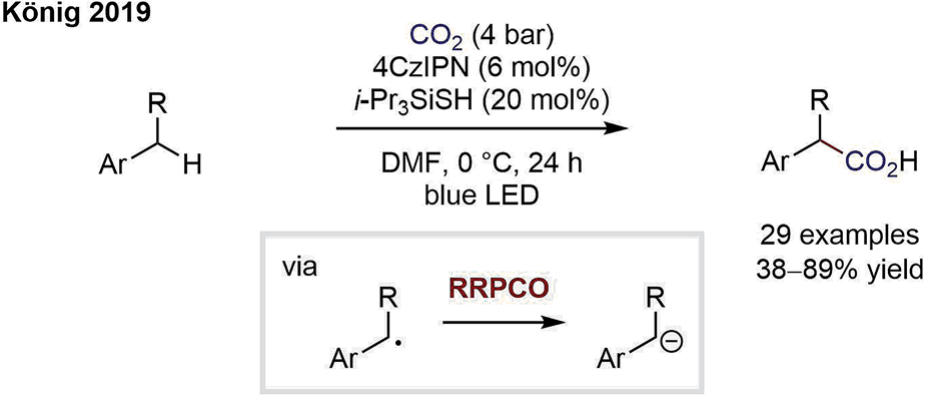
Photocarboxylation of benzylic C–H bonds by König.^13^

The group of König has since reported on an elegant carboxylation of benzylic C–H bonds by merging HAT catalysis with RRPCO (Scheme 8).^13^ First, a thiyl radical is generated *via* oxidation and subsequent deprotonation of a thiol HAT catalyst. The thiyl radical then abstracts a benzylic H atom to generate a benzylic radical and reform the thiol catalyst. As in the previous reaction, the stabilised benzylic radical is then converted to an anion by electron transfer from the reduced photocatalyst and subsequently adds into carbon dioxide to give the carboxylic acid products. The authors not only showed a broad substrate scope but further demonstrated the utility of their protocol by preparing four drug molecules in single steps. Interestingly, even if multiple benzylic C–H sites were present in the molecule, the monocarboxylated products were obtained selectively. This powerful C–H carboxylation clearly underlines the power of RRPCO to convert simple starting materials to high-value products.

### 2.2 Transition metal-mediated radical-transformations

**Scheme 9:**
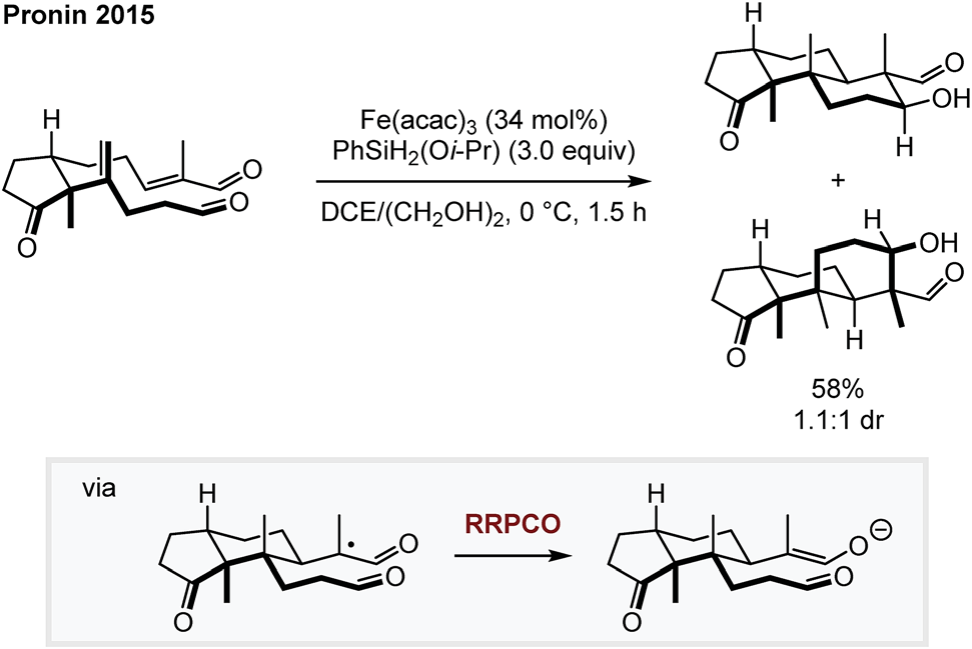
Polycyclisation for the synthesis of terpenoids by Pronin.^14^

The two necessary electron transfer steps in RRPCO reactions can also be accomplished using stoichiometric quantities of external reductants. In 2015, Pronin *et al.* employed the RRPCO concept during their studies of the synthesis of the paxilline indoloterpenoids (Scheme 9).^14^ Here, the RRPCO was elegantly used to construct the complex tricyclic core of this natural product class. Mechanistically, the reaction is initiated by a so called M–H hydrogen atom transfer from the Fe-catalyst to the disubstituted alkene to form a tertiary alkyl radical. This radical undergoes 6-*exo*-trig cyclisation with the pendent α,β-unsaturated aldehyde to form an α-carboxy radical. At this point, the Fe-catalyst promotes the RRPCO to form an enolate intermediate, which subsequently undergoes intramolecular aldol-cyclisation to form the tricyclic product. Although, this polycyclisation did not work in the synthesis of their initial target emindole SB, this example clearly demonstrates the potential of RRPCO in the synthesis of complex structures.

Later, Pronin and co-workers used a RRPCO strategy as a key step in the total synthesis of (–)-nodulisporic acid C and further developed an intermolecular version, which allowed a 14-step total synthesis of the terpenoid forskolin.^15^

**Scheme 10:**
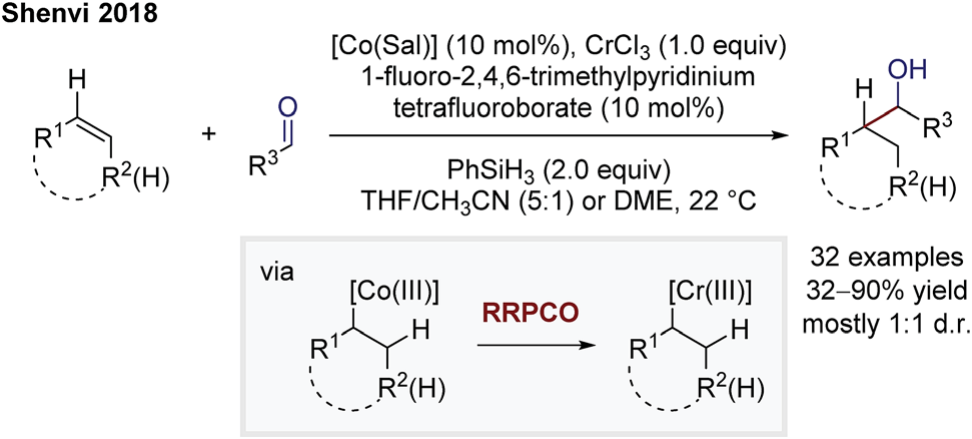
Branch-selective addition of olefins to aliphatic aldehydes by Shenvi.^16^

In 2018, Shenvi and co-workers reported on a branch-selective addition of olefins to aliphatic aldehydes by using a complex mixture consisting of a cobalt catalyst, chromium trichloride, phenylsilane as a superstoichiometric reductant and 1-fluoro-2,4,6-trimethylpyridinium tetrafluoroborate as a substoichiometric oxidant (Scheme 10).^16^ The Co-catalyst undergoes a M–H hydrogen atom transfer to the alkene forming a Co(iii)-alkyl complex. This species transmetallates to a Cr(iii)-alkyl complex enabled by a Cr(ii) complex. This step is considered as the RRPCO step, since this step is only possible beginning from a chromium(ii) species, indicating that an alkyl radical is captured by the chromium(ii) complex to form the Cr(iii)-alkyl species. The *in situ* formed Cr(iii)-alkyl complex is able to add to carbonyls, in this case aliphatic and aromatic aldehydes, to afford alcohols as products.

**Scheme 11:**
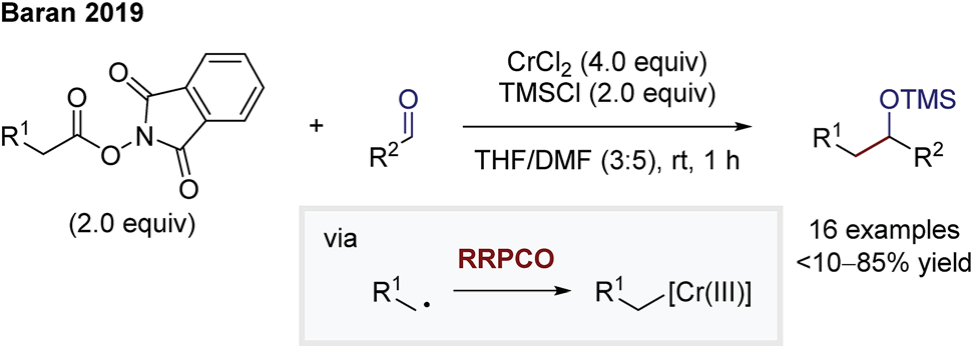
Decarboxylative Nozaki–Hiyama–Kishi reaction by Baran.^17^

In 2019, Baran and co-workers were able to facilitate a decarboxylative Nozaki–Hiyama–Kishi reaction *via* the RRPCO pathway (Scheme 11).^17^ They used redox active esters (RAE) to generate an alkyl radical upon electron transfer from the chromium(ii) species to the RAE. Following the principle of RRPCO, this radical is captured by the excess chromium(ii) salt to form a Cr(iii)-alkyl complex. This nucleophilic species is consequently able to add to aliphatic and aromatic aldehydes as electrophiles. The presence of trimethylsilyl (TMS) chloride in the reaction mixture lead to the isolation of TMS-protected alcohols. Baran and co-workers expanded the scope of the starting materials of traditional alkyl-Nozaki–Hiyama–Kishi reactions to RAE or carboxylic acids.

### 2.3 Dual photoredox-chromium catalysed transformations

**Scheme 12:**
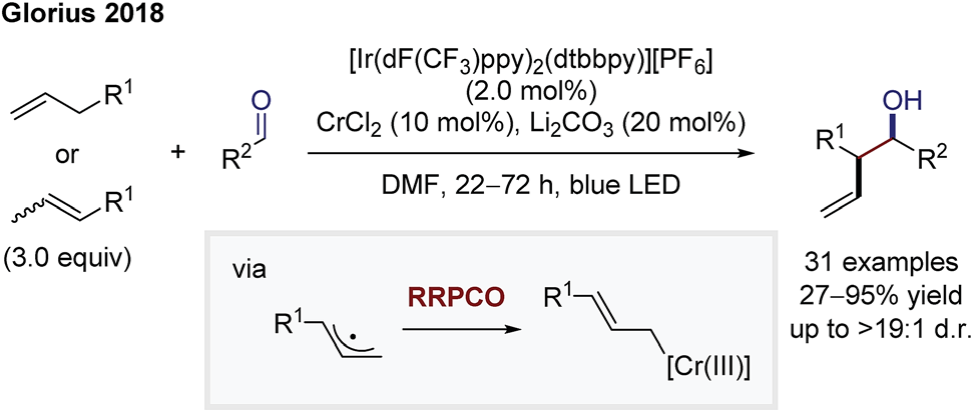
Diastereoselective allylation of aldehydes by Glorius.^19^

The protocols of Shenvi and Baran clearly show the power of chromium(ii) salts in enabling reactions following the RRPCO principle. Nevertheless, the requirement of stoichiometric chromium salts to obtain the desired reactivity is unfortunate, since chromium salts are widely considered to be physiologically hazardous and in case of chromium(ii) expensive and oxygen-sensitive. Furthermore, in enantioselective reactions, (over-)stoichiometric quantities of chiral ligands would be needed, limiting the practicability of such protocols.^18^ In line with this, the groups of Glorius and Kanai published on two protocols, in which these chromium salts could be used catalytically by merging photoredox and chromium catalysis.^19^,^20^ Firstly, Glorius and co-workers showed an allylation of aliphatic and aromatic aldehydes using a dual chromium(ii)- and Ir-photocatalytic ([Ir(dF(CF_3_)ppy)_2_(dtbbpy)][PF_6_]; dF(CF_3_)ppy: 2-(2,4-difluorophenyl)-5-trifluoromethylpyridine) system (Scheme 12).^19^ Both, allyl (hetero-)arenes and β-alkyl styrenes could be employed directly as the Cr(iii)-allyl precursor in this reaction, which represents the first of example of a Nozaki–Hiyama–Kishi reaction based upon a C–H functionalization. Mechanistically, the reaction proceeds *via* oxidation of the allyl compound by the excited photocatalyst, which is followed by rapid proton-abstraction to give an allylic radical. This species undergoes the RRPCO through radical addition to the Cr-catalyst, forming a Cr(iii)-allyl complex. Nucleophilic addition to the aldehyde is facilitated by the Zimmerman-Traxler transition state, thereby providing high diastereomeric ratios of the branched *anti*-products. Finally, reduction of the chromium(iii) species by the reduced photocatalyst closes both catalytic cycles. This example constitutes a rare case of a highly diastereoselective radical reaction and underlines the great potential of RRPCO pathways.

**Scheme 13:**
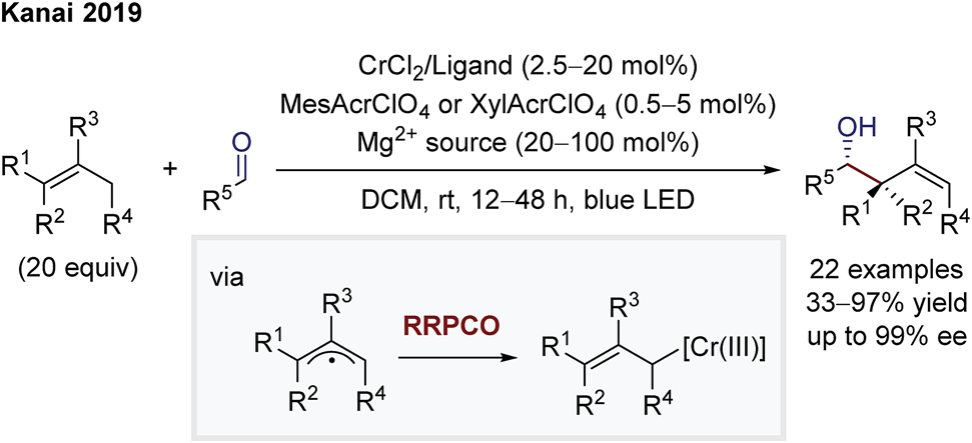
Asymmetric allylation of aldehydes by Kanai.^20^

In 2019, Kanai and co-workers then extended the dual chromium- and photocatalytic system through the addition of chiral ligands to foster an asymmetric allylation of aldehydes (Scheme 13).^20^ The mechanistic scenario is equivalent to that of Glorius. In contrast to Glorius' protocol, Kanai and co-workers used unactivated alkenes as starting materials in combination with a strongly oxidising, organic photocatalyst and obtained high enantioselectivities. In both protocols, the scope is synthetically extremely meaningful, showing a broad functional group tolerance and the utilisation of aliphatic and aromatic aldehydes as electrophiles, providing diastereomerically (Glorius) or diastereo- and enantiomerically (Kanai) enriched products.

## 3. Reductive radical-polar crossover using alkyl halides as electrophiles

**Scheme 14:**
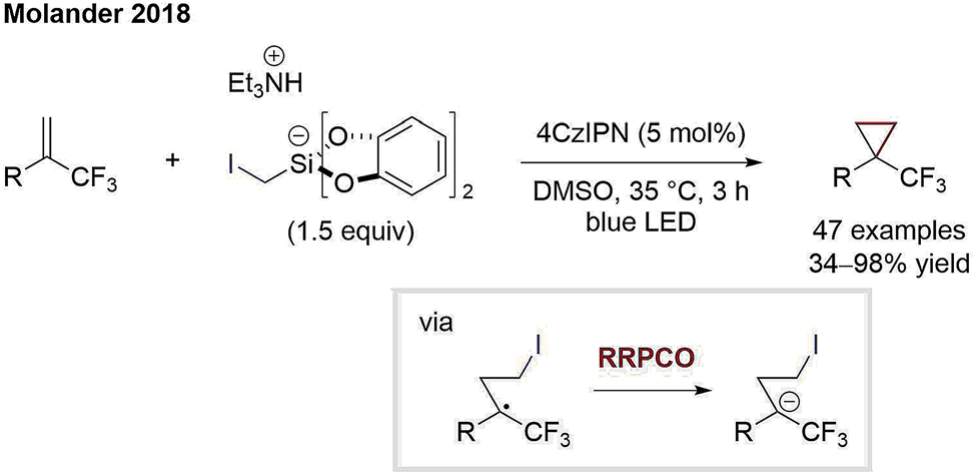
Cyclopropanation by Molander.^21^

The principle of RRPCO was also used to enable nucleophilic substitution reactions using alkyl halides as electrophiles. The Molander group was the first to apply this concept in the context of visible-light photoredox catalysis in 2018. In this work, they described the facile cyclopropanation of alkenes using iodomethyl silicates Scheme 14.^21^ Upon oxidation of the latter, iodomethyl radicals are generated, which can add to α-trifluoromethyl alkenes. The radical formed engages in a RRPCO mediated by the reduced photocatalyst, giving a stabilised anion. Lastly, the nucleophilic substitution takes place, eliminating iodide and closing the cyclopropane ring. Molander and co-workers further adapted the same concept to promote other challenging transformations such as another cyclopropanation process,^22^ the defluorinative alkylation of trifluoromethylalkenes^23^ and an alkylation/cyclisation process with imines.^24^ The highlight of all these transformations is the generation of valuable products based on simple, bench-stable starting materials in a mild and step-economic fashion.

**Scheme 15:**
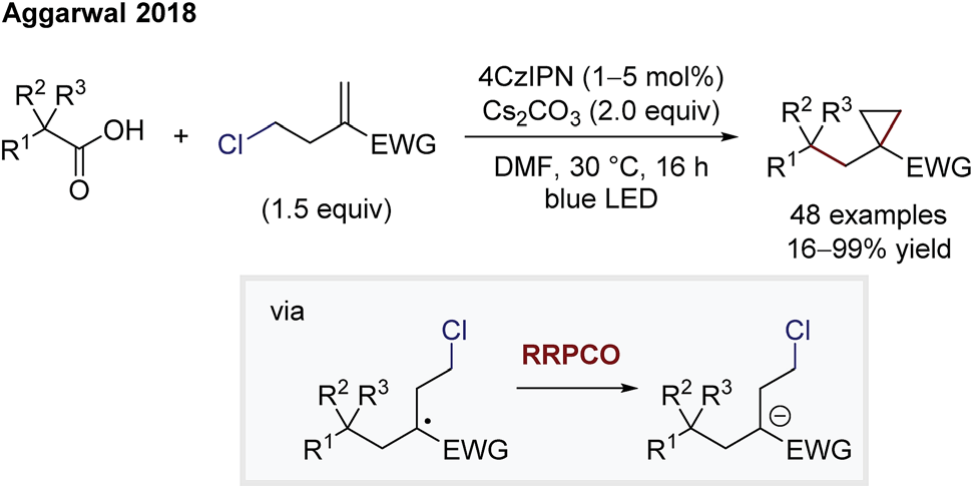
Cyclopropanation by Aggarwal.^25^

Aggarwal and co-workers also showed the utility of RRPCO processes in two transformations, a cyclopropanation (Scheme 15) and a cyclobutane synthesis.^25^,^26^ Both cyclisation processes use the same photocatalyst, 4CzIPN, to facilitate first an oxidation of a radical precursor, and after radical addition to an activated alkene, a reduction of the more stabilised radical. The final electron transfer, here the RRPCO step, generates the required anion that engages in a nucleophilic substitution reaction, leading to the cycloalkane ring closure.

## 4. Conclusions

Reductive radical-polar crossover reactions are initially radical reactions that are converted into traditional nucleophilic reactions by means of a single electron reduction. This reduction is either promoted by a photoredox catalyst or a redox active metal, *e.g.* a chromium(ii) species. Importantly, these transformations allow complex molecules to be assembled in a fast step-economic fashion that would not be possible using either radical or polar chemistry alone. Furthermore, RRPCO enables highly challenging diastereo- or enantioselective radical transformations to be realized. To date, RRPCO reactions have been carried out using carbonyl compounds or alkyl halides/tosylates as electrophiles. Since this field of RRPCO is still in its infancy,^27^ many more transformations using this concept with a wider variety of electrophiles are expected to be developed. Ultimately, we anticipate that this class of reaction will become a widely employed and valuable tool in organic synthesis.

## Conflicts of interest

There are no conflicts to declare.
